# Automatic layout and visualization of biclusters

**DOI:** 10.1186/1748-7188-1-15

**Published:** 2006-09-04

**Authors:** Gregory A Grothaus, Adeel Mufti, TM Murali

**Affiliations:** 1 Department of Computer Science, 660 McBryde Hall, Virginia Polytechnic Institute and State University, Blacksburg VA 24061, USA; 2 Google Inc., 1600 Amphitheater Parkway, Mountain View CA 94043, USA

## Abstract

**Background:**

Biclustering has emerged as a powerful algorithmic tool for analyzing measurements of gene expression. A number of different methods have emerged for computing biclusters in gene expression data. Many of these algorithms may output a very large number of biclusters with varying degrees of overlap. There are no systematic methods that create a two-dimensional layout of the computed biclusters and display overlaps between them.

**Results:**

We develop a novel algorithm for laying out biclusters in a two-dimensional matrix whose rows (respectively, columns) are rows (respectively, columns) of the original dataset. We display each bicluster as a contiguous submatrix in the layout. We allow the layout to have repeated rows and/or columns from the original matrix as required, but we seek a layout of the smallest size. We also develop a web-based search interface for the user to query the genes and samples of interest and visualise the layout of biclusters matching the queries.

**Conclusion:**

We demonstrate the usefulness of our approach on gene expression data for two types of leukaemia and on protein-DNA binding data for two growth conditions in *Saccharomyces cerevisiae*. The software implementing the layout algorithm is available at .

## 1 Background

Measurement of gene expression using DNA microarrays [1,2] have revolutionized biological and medical research. Since gene expression plays an important role in cell differentiation, development, and pathological behavior, computational analysis of DNA microarray data has the potential to assign functions to newly-discovered genes, unravel the structure of biological pathways, and assist in the development of new medicines. Biclustering has emerged as a powerful algorithmic tool for analyzing gene expression data. A bicluster in a gene expression data set is a subset of genes and a subset of conditions with the property that the selected genes are co-expressed in the selected conditions; these genes may not have any coherent patterns of expression in the other conditions in the data set. Biclusters have a number of advantages over clusters computed by more traditional algorithms such as *k*-means and hierarchical clustering [3]. Since a bicluster includes only a subset of genes and samples, it models condition-specific patterns of co-expression. Traditional clusters may miss such patterns since they operate in the space spanned by all the conditions. Further, many biclustering algorithms allow a gene or a sample to participate in multiple biclusters, reflecting the possibility that a gene product may be a member of multiple pathways.

A number of different methods have emerged for computing biclusters in gene expression data [4-16]; two papers survey these techniques [17,18]. These algorithms use different strategies to compute biclusters such as exhaustive enumeration [16,19,20], iterated improvement [5,6], repeated random sampling [11], and expectation maximization [12]. An issue all these algorithms deal with is trying to avoid outputting two or more biclusters with nearly the same set of samples and/or genes. A common approach is to remove a bicluster from the output if it shares a large fraction of genes and/or samples (based on a user-defined threshold) with an already computed bicluster. Another approach replaces the expression values in a bicluster with random values in order to prevent that bicluster from being computed again. In spite of these measures, biclustering algorithms may compute tens, hundreds, or even thousands of biclusters with varying degrees of overlap.

Organising, manipulating, and querying the potentially large number of biclusters computed by these algorithms is a data mining task in itself – one that has not been systematically addressed. In this paper, we develop a novel algorithm for laying out biclusters in a manner that visually reveals overlaps between them. We lay out the biclusters in a two-dimensional matrix whose rows (respectively, columns) are rows (respectively, columns) of the original dataset. We display each bicluster as a contiguous submatrix in the layout. We allow the layout to have repeated rows and/or columns from the original matrix, but we seek a layout of the smallest size. In addition, we develop a web-based search interface that allows the user to query the results for genes and samples of interest and visualise the layout of the biclusters that match the search criteria.

The layout algorithm is general enough to be applied to biclusters computed in real-valued, binary, or categorical data. For instance, the combination of biclustering algorithms and our layout algorithm can be used to analyze measurements of the concentrations of other types of molecules, including proteins and metabolites. We demonstrate our approach on two types of data. First, we compute layouts for biclusters extracted from leukaemia microarray data by the xMotif biclustering algorithm [11,21]. Second, we analyze protein-DNA binding data in *S. cerevisiae *and demonstrate how biclustering in combination with the layout algorithm can visually demonstrate differences in the transcriptional regulatory network that is activated in different growth conditions.

**Figure 1:**
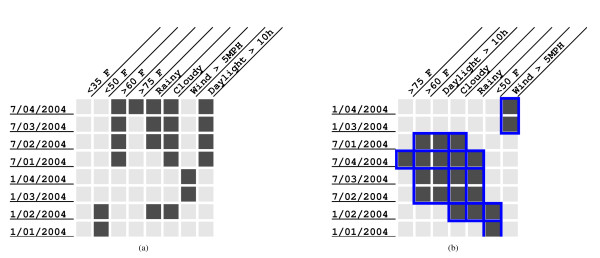
**An example of a bicluster layout for weather data in Blacksburg, VA**. Figure 1(a): a dataset in which rows represent dates and columns represent weather conditions in Blacksburg. Figure 1(b): the layout computed by our algorithm of the seven biclusters in this dataset.

Figure 1 displays a layout computed by our algorithm on a toy binary matrix. Figure 1(a) displays a dataset in which rows represent dates and columns represent weather conditions in Blacksburg, VA, USA. A cell has a one (the cell is drawn shaded) if the weather condition corresponding to the cell's column (e.g., "Rainy" or "> 75°F") is true on the date corresponding to the cell's row. In this dataset, we define a bicluster to be a subset of rows and a subset of columns with the property that the submatrix defined by these rows and columns only contains ones. We computed all the closed biclusters in this binary matrix, i.e., biclusters with the property that every row (respectively, every column) not in the bicluster contains a zero in at least one column (respectively, one row) in the bicluster. In other words, it is not possible to add a row or a column to such a bicluster without introducing a zero. Figure 1(b) displays the layout computed by our algorithm of the seven biclusters in this dataset.

The bicluster layout problem, which we formally define in Section 3.1, is very similar to the hypergraph superstring problem studied by Batzoglou and Istrail in the context of physical mapping of genomes. Batzoglou and Istrail prove that the hypergraph superstring problem is MAX-SNP Hard, i.e., it is computationally intractable to obtain a bicluster layout whose size is smaller than a constant times the optimal size. In this work, we present a heuristic that minimizes the size of the layout well in practice. In the special case when there is a solution involving no repeated rows or columns, the algorithm computes the layout of smallest size. Our algorithm runs in *O*(*mn*^2 ^+ *n*^2 ^log *n*) where *n *is the number of biclusters and *m *is the number of rows and columns in all the biclusters; the running time of the algorithm is independent of the size of the original dataset. We lay out the rows and columns of the biclusters independently. Our algorithm to lay out the columns is similar to a bottom-up hierarchical clustering of the column sets of the biclusters. At each stage, we merge two biclusters if the submatrix induced by them in the original matrix has the "consecutive ones property" (see Section 3.2). Finally we generate the two-dimensional layout by combining the row and column layouts.

## 2 Related work

**Figure 2:**
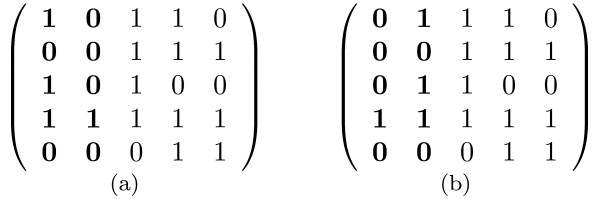
**An illustration of the COP**. Figure 2(a): A matrix that has the COP with the first two columns highlighted. Figure 2(b): Swapping the first two columns of the matrix demonstrates that the matrix has the COP.

A binary matrix has the *Consecutive Ones Property *(COP) for rows if its columns can be permuted such that all the ones in each row are consecutive [22]. See Figure 2 for an example of a matrix with the COP. Determining whether a matrix has the COP and computing the permutation of the columns that proves this property has applications in a number of areas including testing for graph planarity [22] and recognizing interval graphs [22,23]. Booth and Leuker [22] describe a data structure called the PQ tree which they use to represent all legal permutations of column orderings in a matrix with the COP property. They prove that the PQ tree and the correct column permutation can be computed in time linear in the number of ones in the matrix.

Researchers have studied a number of generalizations of the COP problem; however, most of these generalizations are NP-complete or NP-Hard. For example, seeking the column ordering for a non-COP matrix that minimizes the number of gaps between the ones in each row can be reduced to the traveling salesman problem [24]. An important application of generalizations of the COP is physical mapping of chromosomes with probes. We can represent physical mapping data as a binary matrix where the rows represent clones (short overlapping sections of a chromosome), the columns represent DNA probes, and a cell in the matrix has a one if the corresponding probe hybridizes to the corresponding clone. Constructing a physical map of the chromosome is equivalent to finding an ordering of the probes (with probes repeated, if necessary) such that all the probes matching a clone appear consecutively and the total length of the ordering is as small as possible. As mentioned earlier, Batzoglou and Istrail prove that this problem is MAXSNP-Hard [25].

Algorithms for constructing physical maps from hybridization data typically exploit the Lander-Waterman model [26], which assumes that clones are distributed uniformly across the chromosome and that probes are distributed according to independent Poisson processes. Some algorithms make additional domain-specific assumptions [24,25,27-29]. For instance, Batzoglou and Istrail compute an ordering whose length is at most twice the length of the optimal ordering under the requirement that each clone match a probe that does not hybridize to any other clone. None of these algorithms are applicable to our problem since the biclusters we want to lay out may not have the required properties.

## 3 Algorithm

We present our approach in four stages. First, we define some useful notation. Second, we introduce the PQ-tree, a data structure that is fundamental to our approach. Third, we present our layout algorithm. Finally, we discuss its implementation and the web interface to query the computed layout.

### 3.1 Definitions

We denote the input matrix by *D *and use *R *and *C *to denote the set of rows and columns of *D*, respectively. A *layout *L(ℛ, C) of the matrix *D *is a two-dimensional matrix specified as follows:

1. ℛ is the ordered list of rows of L with the property that each element of ℛ is an element of *R*; a row in *R *can appear multiple times in ℛ.

2. C is the ordered list of columns of L with the property that each element of C is an element of *C*; a column in *C *can appear multiple times in L.

3. L_*ij*_, the element in the *i*th row of ℛ and the *j*th column of C is equal to *D*_*i'j'*_, where *i' *is the row of *D *corresponding to the *i*th row of L and *j' *is the column of *D *corresponding to the *j*th column of ℛ.

The *size *of L, is |ℛ||C|. It is appropriate to consider L to be a layout of *D *since L specifies an order for the rows and columns of *D*. We do not require that every row/column of *D *appear in L. In the example in Figure 1(b), the layout does not contain the column titled "< 35F" that is in the original matrix. The layout does not contain any repeated rows or columns either.

Given subsets *R' *⊆ *R *and *C' *⊆ *C*, we define a *bicluster B*(*R'*, *C'*) to be the sub-matrix of *D *spanned by the rows in *R' *and the columns in *C'*. This simple definition is sufficient for this paper. An algorithm that computes biclusters in gene expression data will use a more complex definition relevant to the patterns to be detected. A bicluster *B*(*R'*, *C'*) is *contiguous *in a layout L(ℛ, C) if and only if the elements of *R' *(respectively, *C'*) appear consecutively at least once in ℛ (respectively, L). We say that the layout L(ℛ, C) is *valid *with respect to a set of biclusters *S *if every bicluster *B *∈ *S *is contiguous in L(ℛ, C). For example, the layout in Figure 1(b) is valid with respect to the bicluster ({7/04/2004, 7/03/2004, 7/02/2004}, {> 60F, Daylight > l0 h, Cloudy, Rainy}) since the bicluster spans rows four to six and columns two to five in the layout. We now formally define the *bicluster layout *problem: Given a matrix *D *and a set *S *of biclusters in *D*, find a layout L of *D *such that L is valid with respect to *S *and L has the smallest size among all valid layouts of *D*.

### 3.2 The PQ tree

Booth and Leuker [22] developed a data structure called the PQ tree, which they used to compute a column ordering that proves that that a binary matrix *M *has the COP. To define the PQ tree, it is convenient to reformulate the COP problem as follows: Let *U *be the set of columns of *M*. Let *r *be the number of rows in *M*. For each *i*, 1 ≤ *i *≤ *r*, define the set *S*_*i *_to be the set of columns in *U *that have a one in row *i*. We seek a permutation of the elements of *U *that satisfies *r *restrictions, where *restriction i*, 1 ≤ *i *≤ *r *requires that the elements of *S*_*i *_be consecutive in the permutation.

**Figure 3:**
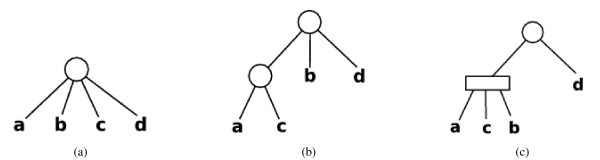
**An example of a PQ-tree**. An example of a PQ tree. Circles represent P nodes and rectangles represent Q nodes. Figure 3(a): Initial PQ tree *T *formed from set {*a*, *b*, *c*, *d*}. Figure 3(b): The PQ tree *T *after the REDUCE(*T*,{*a*, *c*}) operation, requiring that *a *and *c *be consecutive. Figure 3(c): The PQ tree *T *after the REDUCE(*T*,{*b*,*c*}) operation, requiring that *b *and *c *be consecutive. Valid permutations represented by this tree are the sequences *acbd*, *bcad*, *dacb*, and *dbca*.

A PQ tree can represent all legal permutations of *U *that satisfy the restrictions {*S*_*i*_, 1 ≤ *i *≤ *r*}. Each leaf of the PQ tree corresponds to a column in *U*. The PQ tree contains two types of internal nodes: P-nodes and Q-nodes. The children of a P-node can be permuted in any way while still satisfying the restrictions. A valid permutation of the children of a Q-node is either the order in which they appear in the PQ tree or the reversal of this order. A PQ tree supports the REDUCE operation. This operation inserts a restriction *S *into a PQ tree *T*, modifying *T *such that *T *satisfies *S *in addition to all the previous restrictions inserted into *T*. The REDUCE operation fails if there are no legal permutations of *U *that can satisfy *S *and the previously inserted restrictions. The operation takes time linear in |*S*|. Figure 3 displays a PQ tree on four elements {*a*, *b*, *c*, *d*} after two REDUCE operations: REDUCE(*T*,{*a*, *c*}) and REDUCE(*T*,{*b*, *c*}). Inserting the restriction {*c*, *d*} into the tree next will result in a failed REDUCE operation.

To solve the COP problem, start with an empty PQ tree *T*. For each *i*, 1 ≤ *i *≤ *r*, invoke the operation reduce(*T*, *S*_*i*_). To obtain an ordering that satisfies the restrictions, perform a breadth-first traversal of *T *starting at the root. At each internal node of *T*, visit the children of the node in any order that is valid for the type of the node. At a leaf node of *T*, append the column corresponding to the leaf to the required ordering.

### 3.3 The bicluster layout algorithm

We are now ready to describe our algorithm for the bicluster layout problem. To minimize the size of L, we can minimize the length of ℛ and the length of C independently. Therefore, we construct the layout L by determining ℛ and C independently. In the rest of this section, we describe the algorithm to construct C, the ordered list of the columns in the layout L. We can compute ℛ, the ordered list of rows in the layout, analogously.

We describe the algorithm in two stages. We first transform the problem of constructing C to a generalization of the COP problem. We then present an algorithm to solve this transformed problem. This transformation allows us to describe our algorithm in terms of operations on PQ trees. The PQ tree cannot solve this generalization directly since the matrix we construct may not have the COP.

We start by constructing a new binary matrix *M *that represents the columns of the biclusters in *S*. Each column on *M *corresponds to a column of the input matrix *D*. *M *contains one row for each bicluster in *S*; thus, *M *has *n *rows. The entry *M*_*ij *_is 1 if the *i*th bicluster in *S *contains the column *j *in *D*; otherwise, *M*_*ij *_is 0. We can now reformulate the problem of constructing C as follows: find the shortest linear ordering C of the columns of *M *such that C can contain repeated columns of *M *and for every row of *M*, the columns containing the ones in that row appear consecutively at least once in C.

Before describing the algorithm, we define some more notation. The leaves of each PQ tree constructed by the algorithm correspond to a subset of the columns of *M*. We use *C*_*T *_to denote the set of columns in a PQ tree *T*. Given two PQ trees *T *and *T'*, let *σ*(*T*, *T'*) denote the set similarity $\frac{\left|C_{T}\cap C_{T^{\prime }}\right|}{\left|C_{T}\cup C_{T^{\prime }}\right|}$ between the columns in *T *and *T'*. Our algorithm executes the following steps:

1. For each row *i *of *M*, 1 ≤ *i *≤ *n*, construct a PQ tree *T*_*i *_and insert the restriction corresponding to row *i *of M into *T*_*i*_. Let T be the set of these *n *PQ trees.

2. For every pair 1 ≤ *i *≤ *j *≤ *n*, compute the set similarity *σ*(*T*_*i*_, *T*_*j*_).

3. Compute Σ, the list of values in {*σ*(*T*_*i*_, *T*_*J*_), 1 ≤ *i *≤ *j *≤ *n*} sorted in descending order.

4. Repeat the following steps until Σ is empty:

(a) Remove the largest element from Σ. Let *T *and *T' *be the PQ trees in T with this similarity value.

(b) Set *T" *= *T*.

(c) For each restriction *r *inserted into *T'*, invoke the operation REDUCE(*T"*, *r*). If any reduce operation fails, go to Step 4a.

(d) Delete *T *and *T' *from T.

(e) For each tree *U *∈ T, insert *σ*(*U*, *T"*) into Σ.

(f) Insert *T" *into T.

5. For each PQ tree *T *in T, traverse *T *to compute a valid permutation of the columns in *C*_*T*_.

6. Output the column layout formed by concatenating (in any order) the permutations computed in Step 5.

The algorithm starts by storing each row of *M *in a separate PQ tree in the set T (Step 1). Next, the algorithm performs a series of REDUCE operations to hierarchically cluster the rows of *M*. Inductively, the restrictions inserted into each PQ tree in T correspond to a set of rows of *M *with the property that the submatrix of *M *spanned by these rows has the COP. To decide which two sets of rows to merge next, in Step 4a, the algorithm picks the two PQ trees *T *and *T' *in T that are the most similar and attempts to merge them. To effect the merger, the algorithm adds the restrictions added to one of these PQ trees to the other PQ tree (Step 4c). If this step succeeds, the algorithm deletes *T *and *T' *from T, inserts the similarities between the new PQ tree *T" *and each of the remaining PQ trees in T into Σ, and inserts *T" *into T (Steps 4d–4f). In Step 4c, the failure of a REDUCE operation means that the restrictions in *T *are not compatible with the restrictions imposed by *T'*. Hence, the submatrix of *M *induced by the union of rows in *T *and in *T' *does not have the COP. An example of such a situation is when *T *corresponds to the tree in Figure 3(c) and *T' *contains the restriction {*c*, *d*}. In this case, the algorithm aborts the merger of *T *and *T' *and moves on to the next most similar pair of PQ trees. Due to such conflicts, T may contain more than one PQ tree when the algorithm completes. Finally, generating the required layout is a simple matter of traversing each PQ tree in T (Step 5) as described in Section 3.2 and concatenating the resulting permutations into a single order (Step 6). A column of *M *appears as many times in this order as there are PQ trees in T that include this column.

We now analyze the running time of the algorithm. Let *m *be the number of ones in the matrix *M*. As stated earlier, the number of biclusters in the input is *n*. In Step 1, computing the PQ trees takes *O*(*m*) time. Computing the similarity between a pair of PQ trees takes *O*(*c*) time, where *c *is the number of columns of *M*. Thus, in Steps 2 and 3, computing and sorting the *O*(*n*^2^) similarity values takes *O*(*cn*^2 ^+ *n*^2 ^log *n*) time. We execute Step 4 *O*(*n*^2^) times. The running time of each iteration is proportional to the size of the new PQ tree constructed. A naive upper bound on this size is *m*, the total number of columns in all the biclusters. Hence, the total running time of Step 4 is *O*(*mn*^2^). Finally, traversing all the PQ trees in T and concatenating the permutations takes *O*(*m*) time. Keeping in mind that *c *≤ *m*, the total running time of the algorithm is *O*(*mn*^2 ^+ *n*^2 ^log *n*). The space used by the algorithm is *O*(*m *+ *n*^2^), with *O*(*m*) space taken to store all the biclusters and the PQ trees and *O*(*n*^2^) required for Σ, the sorted list of similarities.

### 3.4 Implementation and web interface

We implemented the layout algorithm in C++ and tested it on a 2.8 GHz Pentium computer running the Fedora Core 3 operating system. Our software contains two executable programs. The first executable, layout, implements the layout algorithm. It takes a text file describing the biclusters as input and outputs the layout in a simple textual format that specifies the order of the rows and columns in the layout and the corners of each bicluster in the layout. The second executable, drawlayout, uses the computed layout and the original data set as input and produces an image corresponding to the layout.

If the input data contains a large number of biclusters, the layout may contain too many rows and/or columns for the user to navigate with ease. To alleviate this problem, we have also developed a simple web-based interface that allows the user to upload a file containing computed biclusters and a file containing the original data, and query the layout with the names of rows and columns. The interface invokes layout and drawlayout on the biclusters that contain the query rows/columns and highlights the matching biclusters, rows, and columns in the resulting layout. The interface allows the user to specify whether the data is real-valued or binary, whether the layout should contain only the matching biclusters, and whether the query should be a conjunction or disjunction of the search terms.

## 4 Experimental results

We present results for three types of data. We first evaluated our method on synthetic datasets. Next, we considered a binary data set encoding results of ChIP-on-chip experiments in *S. cerevisiae*. Finally, we used our method on gene expression data to distinguish differences between two types of leukaemia.

### 4.1 Synthetic data

We created synthetic datasets with different numbers of rows and columns. For each dataset, we generated biclusters by sampling subsets of rows and columns. For this experiment, we randomly generated the number of rows and columns and identifiers for the rows and columns; we did not need to generate values for the cells of the matrices. For each set of biclusters, we recorded the time required to run our layout algorithm and the number of rows and columns in the computed layout. For each layout, we estimated the *efficiency *of the layout as the ratio of the size of the layout to the size of the dataset. Lower values of efficiency are better than higher values, since they indicate that the algorithm is able to exploit overlaps between biclusters. For each choice of number of rows in the dataset, number of columns in the dataset, and number of biclusters, we averaged the results for 100 runs. Tables 1 and 2 display our results. Efficiency values may be less than one, e.g., when some rows or columns in the dataset do not belong to any bicluster.

**Table 1:** Execution times (in seconds) for the layout algorithm on synthetic matrices

#biclusters	#rows + #columns in the dataset				
	10	30	50	70	90

20	0.168	0.328	0.462	0.52	0.532
40	1.23	2.514	3.046	3.574	4.008
60	4.074	7.992	11.238	11.71	12.81
80	9.484	19.586	25.546	29.652	29.446
100	17.982	37.966	48.418	50.916	56.112

**Table 2:** Efficiency values for the layout algorithm on synthetic matrices.

# biclusters	#rows + #columns in the dataset				
	10	30	50	70	90

20	0.184	0.842	1.316	1.254	1.428
40	0.304	1.16	1.632	2.04	2.074
60	0.398	1.496	2.262	2.26	2.508
80	0.512	1.65	2.358	2.726	2.698
100	0.48	1.808	2.582	2.686	2.996

### 4.2 Transcriptional regulation in S. cerevisiae

To demonstrate the ability of our visualization algorithm to highlight differences between biclusters in similar datasets, we analyzed datasets of transcriptional regulation in two experimental conditions in *S. cerevisiae *[30,31]. Each dataset is a binary matrix whose columns represent transcription factors and whose rows represent genes in *S. cerevisiae*. A matrix entry contains a one if a ChIP-on-chip experiment indicates that the transcription factor binds to the promoter of the gene with a p-value at most 0.001. An important problem that arises in the analysis of this data is determining if a set of genes are collectively regulated by a set of transcription factors and whether this combinatorial regulation changes when the cell is exposed to stress. Although ChIP-on-chip data is noisy and significant effort may be needed to clean it up, the analysis we present next demonstrates that a combination of biclustering and our layout algorithm yields biologically useful results.

**Figure 4:**
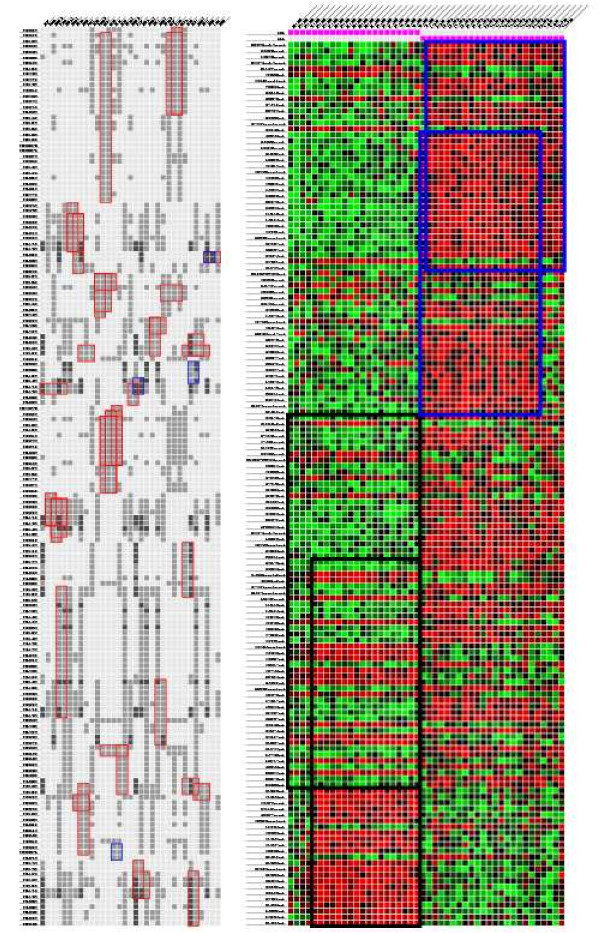
**Bicluster layouts**. Visualizations of the layouts computed by our algorithm. Since the layout may contain repeated rows and columns, a bicluster may appear at multiple locations in the layout. We only highlight only one occurrence of each bicluster. The layout on the left displays biclusters representing combinatorial control of transcription in *S. cerevisiae*. The layout on the right displays biclusters in gene expression data for ALL and AML.

The two protein-DNA datasets we study correspond to the growth of *S. cerevisiae *cells in rich medium [31] and to growth under exposure to rapamycin [30], a condition that mimics nutrient starvation. We restricted our attention to transcription factors studied in both papers. We ran our implementation of the *Apriori *algorithm [32] that computes closed biclusters (as defined in Section 1) on both these datasets, applied our layout algorithm on biclusters with at least two genes and at least two transcription factors, and obtained the layout in Figure 4(a). Biclusters obtained from the data under growth in rich medium are shown as blue boxes and rapamycin-induced biclusters are shown as red boxes. A cell in the figure is dark grey (respectively, light grey) if the transcription factor binds to the gene's promoter in both (respectively, one) condition. The image strikingly demonstrates that under exposure to rapamycin, the transcriptional regulatory network activated in the cell is very different from the network activated under growth in rich medium. The rich medium data contains only four biclusters involving these transcription factors while the rapamycin data contains 38 biclusters. We conclude that very few genes are co-regulated by the same set of transcription factors in both conditions.

To illustrate the use of our web interface, we used it to search for biclusters that included the transcription factors RTG3 and GLN3. RTG3 is a transcription factor that forms a complex with RTG1 to activate the retrograde (RTG) and target of rapamycin (TOR) pathways [33,34]. GLN3 encodes a transcription factor that is phosphorylated and localised to the cytoplasm when the cell is grown in nitrogen-rich media.

**Figure 5:**
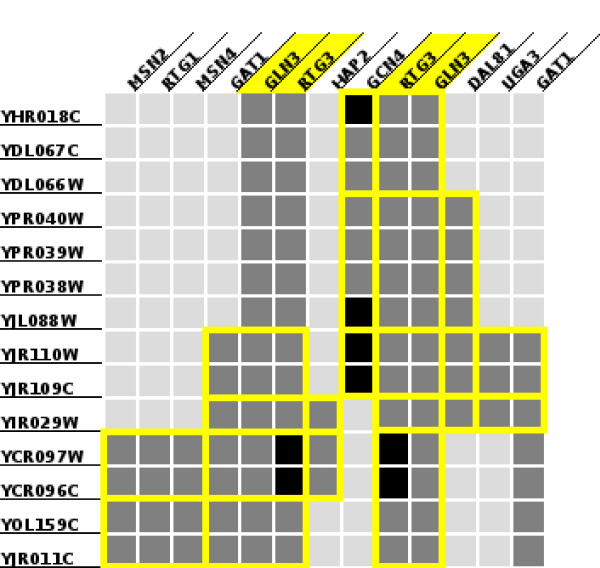
**Genes combinatorially controlled by GLN3 and RTG3**. A layout of nine biclusters of genes combinatorially controlled by GLN3 and RTG3 under exposure to rapamycin.

Rapamycin treatment can induce the dephosphorylation and subsequent activation of GLN3 [35]. Figure 5 displays the layout of all the biclusters containing these two transcription factors. We note that all but one bicluster also includes either the transcription factor GAT1 or the transcription factor GCN4. GAT1 is a transcriptional activator of genes involved in nitrogen catabolite repression; the activity and localization of these genes is regulated by nitrogen limitation. GCN4 is another transcription activator that is a master regulator of gene expression during amino acid starvation in *S. cerevisiae *and is activated in multiple stress responses [36]. Thus, it is not surprising that GAT1 and GCN4 co-regulate genes with GLN3 and RTG3. The functional annotations of the set of nine genes targeted by GCN4, GLN3, and RTG3 is enriched in the Gene Ontology biological process "glutamine family amino acid biosynthesis" (*p*-value of 2 × 10^-8^, based on the hypergeometric distribution), indicating that this pathway may be activated by the three transcription factors upon rapamycin treatment.

### 4.3 Classification of leukaemias

Golub et al. [37] studied global expression patterns of 45 patients diagnosed with Acute Lymphoblastic Leukaemia (ALL) and 27 patients diagnosed with Acute Myeloid Leukaemia (AML). We ran the xMotif algorithm [11,21] to compute biclusters in this dataset. We ensured that computed biclusters contain samples from at most one class. We selected four representative biclusters from the results to visualize. Figure 4(b) displays the layout. Each column corresponds to a sample; the two columns at the top with purple cells indicate the type of leukaemia. We map the expression values of each gene into a range from green to red, with green (respectively, red) corresponding to the smallest (respectively, largest) expression value of that gene. The biclusters outlined in black correspond to AML samples and those outlined in blue to ALL samples. This layout visually highlights similarities and differences between the biclusters found in samples for the same and for different types of leukaemia. We have used such biclusters as the basis for constructing a classifier that distinguishes between different diseases and tissues (Grothaus and Murali, in preparation).

## 5 Conclusion

The biomedical community has access to large quantities of publicly-available gene expression datasets. Biclustering has emerged as a powerful methodology for analyzing these datasets. In this paper, we have introduced a novel algorithm for laying out biclusters in a two-dimensional matrix so as to reveal the overlaps and relationships between the biclusters. The algorithm performs efficiently in practice. We have demonstrated the applicability of the algorithm to three important problems in bioinformatics using both binary and real-valued data. An easy-to-use web interface distributed with the layout software allows the user to query and navigate layouts that are too large to study manually. Biclustering is useful not just for processing gene expression data but for any dataset that measures the relationships between two different types of data, e.g., genes and functions; microRNAs and their target mRNAs; and genes and diseases. Thus, our algorithm has the potential to be useful for a wide variety of bioinformatic applications.

## Authors' contributions

TMM posed the problem to GG. GG developed and implemented the algorithm and performed the experiments with guidance from TMM. AM implemented the web interface. GG and TMM wrote the paper.
